# The Impact of Maternal High-Fat Diet on Bone Microarchitecture in Offspring

**DOI:** 10.3389/fnut.2021.730037

**Published:** 2021-08-30

**Authors:** Emma J. Buckels, Scott M. Bolam, Mei Lin Tay, Brya G. Matthews

**Affiliations:** ^1^Department of Molecular Medicine and Pathology, School of Medical Sciences, University of Auckland, Auckland, New Zealand; ^2^Department of Surgery, School of Medicine, University of Auckland, Auckland, New Zealand; ^3^Department of Orthopaedic Surgery, Auckland City Hospital, Auckland, New Zealand

**Keywords:** early life nutrition, maternal obesity, developmental origins of health and disease (DOHaD), osteoporosis, osteoblast, osteoclast, bone marrow adipocytes, skeletal development

## Abstract

The incidence of obesity in women of reproductive age has significantly increased over the past 100 years. There is a well-established connection between maternal obesity during pregnancy and an increased risk of developing non-communicable cardiometabolic diseases in her offspring. This mini-review focuses on evidence examining the effect of maternal high-fat diet (HFD) on skeletal development and bone health in later life in offspring. The majority of rodent studies indicate that maternal HFD generally negatively affects both embryonic bone development and bone volume in adult animals. Details surrounding the mechanisms of action that drive changes in the skeleton in offspring remain unclear, although numerous studies suggest that some effects are sex-specific. Human studies in this area are limited but also suggest that HFD during pregnancy may impair bone formation and increase fracture risk during childhood. Given the consequences of low bone mass and deranged bone microarchitecture for offspring, advances in our understanding of the developmental origins of bone health is critical in the battle against osteoporosis.

## Introduction

The prevalence of obesity over the past 100 years has dramatically increased, with obesity identified as the most common metabolic disorder. Globally, an estimated 600 million adults were obese (body mass index ≥ 30 kg/m^2^), and 1.9 billion adults were overweight in 2015 (body mass index 25–30 kg/m^2^) (1). The prevalence of obesity is expected to reach 1.12 billion individuals by 2030 (2). Obese individuals have an increased risk of morbidity from type 2 diabetes mellitus (T2DM), cardiovascular disease, specific cancers, and osteoarthritis (3).

The incidence of obesity in women of reproductive age has also increased. Maternal obesity is a significant risk factor for maternal, fetal, and neonatal morbidities, including miscarriage, preterm delivery, hypertension, pre-eclampsia, and gestational diabetes (4–6). Research in the field of developmental origins of health and disease (DOHaD) has highlighted that maternal obesity during pregnancy predisposes offspring to develop obesity and other non-communicable diseases, including T2DM, hypertension, and cardiovascular disease, later in adulthood (7). Paternal obesity also increases the risk of developing non-communicable diseases in offspring (8–10), and both maternal and paternal obesity have transgenerational effects on subsequent generations via epigenetic effects on the germline (10–12). Thus, obesity and its related comorbidities represent an increasing burden on healthcare systems.

Although not fully understood, the effect of maternal obesity on the development of various organs and tissues such as the brain, liver, kidney, endocrine pancreas, and skeletal muscle and their structure and function have been well-researched with the aid of animal models. In these models, maternal obesity is induced via a high-fat diet (HFD) (13, 14). Recently the effects of maternal HFD on bone mass and strength in offspring and the risk of developing osteoporosis later in life have been researched. This mini-review will discuss evidence that maternal HFD-induced obesity affects bone development and microarchitecture, focusing on recent advancements using rodent models, and will discuss the potential mechanisms involved.

## Early Bone Development and Impact Later in Life

The skeleton develops *in utero* from mesenchymal condensations. Most of the skeleton forms via a process known as endochondral ossification: initially, a cartilaginous template forms, which is later progressively replaced by mineralized bone matrix. Cartilaginous growth plates continue to control the longitudinal growth of bones throughout neonatal and childhood growth, while the overall bone shape, mineralization, and microarchitecture are determined by the balance of bone formation by osteoblasts and bone resorption by osteoclasts in different locations. Longitudinal skeletal growth continues until the late teens in humans, ending when the growth plates fuse. However, bone mineral density (BMD) continues to increase slowly until peak bone mass is reached in the mid-20s to early-30s. Rodents continue to grow slowly for a longer portion of their life, but mice reach peak bone mass at ~12-weeks of age. Bone mass begins to decline as bone resorption outpaces bone formation as we age. In women, there is a dramatic period of bone loss following menopause when reductions in sex hormone levels affect homeostasis. Skeletal size, BMD, and bone microarchitecture are largely determined by genetics, with up to 85% of the variation in BMD explained by genetic factors (15). However, various non-genetic factors also influence both bone accrual during growth and bone loss in later life. Nutrition is a major factor influencing both growth and bone mass and can have effects at all life stages. Exercise, or the effect of loading on the skeleton, plays a major role in bone accrual and retention.

Osteoporosis is characterized by low BMD and a high risk of fracture, and affects one-half of elderly women and about one-fifth of elderly men. While osteoporosis is considered a disease of aging, early life events and a failure to achieve maximal peak bone mass determined by an individual's genetics can significantly impact future osteoporosis risk. One line of evidence for long-term impacts of events earlier in life comes from studies in athletes. Baseball pitchers who develop larger, stronger bones in their throwing arm during early adulthood can retain better bone structure in this arm for 50 years following retirement from the game (16). Various drugs are available that effectively reduce fracture risk in people with osteoporosis; however, the majority are antiresorptive therapies that prevent further bone loss but do not enable the replacement of bone already lost (17). Anabolic treatments restore bone microarchitecture to some degree, but are expensive biologics so not available to everyone. Understanding risk factors for low bone mass and identifying people at high risk of fracture are important for preventing fracture-related morbidity and mortality in aging populations.

## Effect of Maternal HFD on Bone in Rodents

Thirteen rodent studies considered the effects of maternal HFD on offspring bone development (Table 1). The majority of studies implemented the maternal HFD regime before mating (4–15 weeks before conception) and continued through pregnancy and lactation. However, in three studies, the maternal HFD-feeding window was exclusively during pregnancy and lactation and exclusively during lactation in one study.

**Table 1:** Studies investigating the effect of maternal HFD on offspring bone properties.

**Dietary details^a^**	**Animal strain**	**Dietary intervention period**	**Offspring age**	**Main findings in maternal HFD vs. CD**	**Proposed mechanism(s) of action of maternal HFD on offspring bone properties**	**References**
**Fetal**						
HFD (45% fat) CD (17% fat)	C57BL/6J mice	8 weeks before mating and pregnancy	E17.5	Decreased total bone volume and bone mineralisation, increased senescence markers, pro-inflammatory cytokines, and chemokines in calvarial osteoblasts.	Maternal HFD promotes osteo-progenitor senescence and expression of pro-inflammatory factors, which could impair fetal skeletal development.	(18)
HFD (45% fat) CD (17% fat)	Sprague-Dawley rats	10 weeks before mating and pregnancy	E18.5	Decreased bone formation and ossification in calvaria and vertebrae, and decreased potential for calvarial osteoblast differentiation.	Demonstrate decreased osteogenic differentiation via hypermethylation and decreased expression of HoxA10.	(19)
HFD (42% fat) CD (17% fat)	Sprague-Dawley rats	12 weeks before mating and pregnancy	E18.5	Increased expression of p53/p21-mediated cell senescence signaling-related genes and proteins in calvarial osteoblasts.	Hypothesis: increased cell senescence may result in decreased glucose metabolism and cell differentiation.	(20)
HFD (60% fat) CD (18% fat)	C57BL/6J mice	4 weeks before mating and pregnancy	E19	Decreased body length, total bone volume, long bone lengths, and BMD. Some effects are ameliorated by maternal antioxidant supplementation.	Hypothesis: increased oxidative stress leads to placental vascular damage and impaired osteogenic fetal signaling pathways.	(21)
**Postnatal**						
HFD (60% fat) CD (10% fat)	Sprague-Dawley rats	Pregnancy and lactation only	P1 and P21	Increased Tb.BV/TV at P1 and P21.	Hypothesis: increased bone volume is driven by increased osteoblast activity.	(22)
			5 and 15 weeks	Decreased femur length, Tb.BV/TV, at 15 weeks (males only). Increased osteoclast number and surface, and osteoclastogenesis *ex vivo*.	Demonstrate increased osteoclast activity in 15-week males.	(22)
HFD (41% fat) CD (17 % fat)	Wistar rats	10 weeks before mating, pregnancy, and lactation	P28	Increased BMD and fatty acid content in the femur at P28.	None.	(23)
			12 weeks	BMD, femoral bone strength and fatty acid content not different.	None.	(23)
HFD (45% fat) CD (18% fat)	C57BL/6J mice	6 weeks before mating, pregnancy, and lactation	14 and 26 weeks	Increased femoral Tb.BV/TV at 14 weeks, not different at 26 weeks. No difference in bone strength. MAR increased in males at 14 weeks.	May be sexually dimorphic mechanisms involved. Males had higher MAR and lower osteoclast activity at 14 weeks.	(24)
HFD (60%) CD (10%)	C57BL/6J mice	11–15 weeks before mating and during pregnancy and lactation	28 weeks (F1 and F2 offspring)	Decreased Tb.BV/TV and BMD in tibia in F1 and F2 female offspring. No changes in males.	None.	(25)
HFD (60% fat) CD (18% fat)	C57BL/6J mice	4 weeks before mating and during pregnancy and lactation	26 and 52 weeks (females only)	Decreased femoral BMD at 26 weeks, increased Tb.Sp at 52 weeks.	None.	(26)
**Post-weaning crossover diet studies**						
HFD (45% fat) CD (7% fat)	C57BL/6J mice	Pregnancy and lactation. Four groups at weaning: CD/CD, CD/HFD, HFD/CD, HFD/HFD	6 weeks	Increased femoral length, bone volume, and cortical thickness (males only); changes were amplified HFD/HFD. MGP expression negatively correlated with bone volume.	None.	(27)
HFD (45% fat) CD (17 % fat)	C57BL/6J mice	8 weeks before mating and during pregnancy and lactation. Four groups at weaning (as above)	17 weeks	Decreased Tb.BV/TV in all male HFD groups, increased CSA and medullary area in HFD/CD males.	Increased expression of senescence-related proteins in both ages. Early effects after maternal HFD persist into adulthood.	(18)
HFD (60% fat) CD (14% fat)	C57BL/6J mice	Lactation only. Weaned onto CD, 4 groups at 12 weeks (as above)	24 weeks	Decreased Tb.BV/TV in HFD/HFD only (males, females not analyzed). Lactational HFD increased bone marrow adiposity, further amplified in HFD/HFD.	Hypothesis: BMSCs are more committed to a pro-adipogenic lineage, resulting in greater bone marrow adiposity and decreased bone mass.	(28)
HFD (43% fat) CD (14% fat)	C57BL/6J mice	7 weeks before mating and during pregnancy and lactation. Three groups at weaning (no CD/HFD)	30 weeks	Femurs shorter in HFD/HFD, no difference in Tb.BV/TV. Increased number of bone marrow adipocytes and diameter of adipocytes (females only) in HFD/HFD vs. CD/CD.	None.	(29)
HFD (45% fat) CD (7% fat)	C57BL/6J mice	Pregnancy and lactation. Four groups at weaning (as above)	30 weeks	Femoral Tb.BV/TV decreased in HFD/HFD males. Vertebral Tb.BV/TV decreased in HFD/CD males. No changes in females.	None.	(30)

a *All shown as % kcal from fat*.

*BMSC, bone marrow stromal cell; BMD, bone mineral density; CD, control diet; CSA, cross section area; E, embryonic day; F1/2, first/second generation offspring; HFD, high-fat diet; HoxA10, homeodomain-containing factor A10; MAR, mineral apposition rate; MGP, matrix gla protein; P, postnatal day; Tb.BV/TV, trabecular bone volume fraction; Tb.Sp, trabecular separation*.

### Fetal and Neonatal Offspring

Offspring of dams fed a maternal HFD before and during pregnancy have evidence of skeletal developmental delay in late-gestation with decreased bone formation, bone volume, and BMD (18–21). Chen et al. have demonstrated that maternal HFD promotes cellular senescence in fetal calvarial osteoblasts cells, potentially suppressing fetal bone formation (18–20).

In both newborns and weanlings, exposure to maternal HFD resulted in increased total bone mass, BMD, and trabecular bone volume in long bones (22, 23). This phenotype is likely the result of increased osteoblast activity, as bone modeling is most active over this period of rapid growth (31). Increased bone mass in weanlings may be an indirect effect of maternal HFD, as these offspring consume more milk, and the milk consumed has a higher energy content compared to dams fed control diet (CD) (22). Additionally, Miotto et al. found higher concentrations of monounsaturated fatty acids in the long bones of offspring exposed to maternal HFD, which is likely to reflect the diet consumed by the dams; these lipid stores may have supported rapid bone growth (23).

### Adult Offspring

From early adulthood, a pattern of sustained bone loss in offspring of dams fed HFD is reported. In most studies, offspring exposed to maternal HFD have reduced BMD and bone volume in long bones and vertebrae from as early as 8-weeks of age, which persisted over their lifetime (18, 21–23, 25, 30). Hafner et al. found that maternal HFD during lactation alone was sufficient to increase bone marrow adiposity (28). Maternal HFD decreased trabecular bone parameters in offspring (18, 21–23, 25, 30). However, this effect was not observed in all studies; two studies found increased femoral bone trabecular volume and increased cortical thickness following maternal HFD (24, 27). Notably, these two studies analyzed offspring who were young adults, as opposed to the studies that found decreased bone volume in mice who were considerably older.

Several studies demonstrated sex-specific variation in the effect of maternal HFD, with males more likely to exhibit a bone phenotype than females (18, 22, 24). Only one study exclusively found changes in bones in females following maternal HFD (25). Therefore, maternal HFD most likely has a sexually dimorphic effect on the skeleton of offspring.

### Multigenerational Effects of Maternal HFD

Maternal HFD can have multigenerational effects on bone in offspring and grand-offspring. Harasymowicz et al. found decreased trabecular bone volume and BMD in F1 and F2 generations, even with no additional exposure to HFD in either generation (25).

### Postnatal Exposure to HFD

A post-weaning HFD, or a “second hit,” has the potential to amplify the effects of maternal HFD (32). Three studies that investigated continued feeding of HFD in offspring after weaning found that post-weaning HFD further decreased trabecular bone volume (28, 30) or increased bone-marrow adiposity (28, 29), compared to exposure to maternal HFD alone.

## Effect of Maternal Obesity and HFD on Bone in Humans

Several studies have specifically addressed whether maternal obesity or maternal HFD during pregnancy affects bone development in offspring, both *in utero* and post-partum (33). Longitudinal studies show that obese mothers have babies with increased body length, whole-body bone area, and mineral content (34–36), but maternal diet was not reported. Two studies demonstrated that mothers consuming a high-fat “Western diet” during pregnancy, defined as a diet high in meat, processed food, and saturated fat, have children with lower whole-body bone area, bone mineral content, and BMD, compared with children of mothers on low-fat “prudent diets” during pregnancy, defined as a diet high in fruits, vegetables, grains and low-fat dairy products (37, 38). Interestingly, offspring of mothers in the Danish National Birth Cohort who consumed a Western diet had a significantly increased risk of fracture between birth and 16-years of age (39). None of these studies reported maternal BMI related to study groups. Due to the paucity of human data (33), it is unclear whether maternal obesity in the absence of HFD, maternal HFD in the absence of obesity, or any other dietary conditions of over-nutrition with or without maternal obesity affects the skeletal phenotype in human offspring.

## Mechanisms of Action

**Figure 1:**
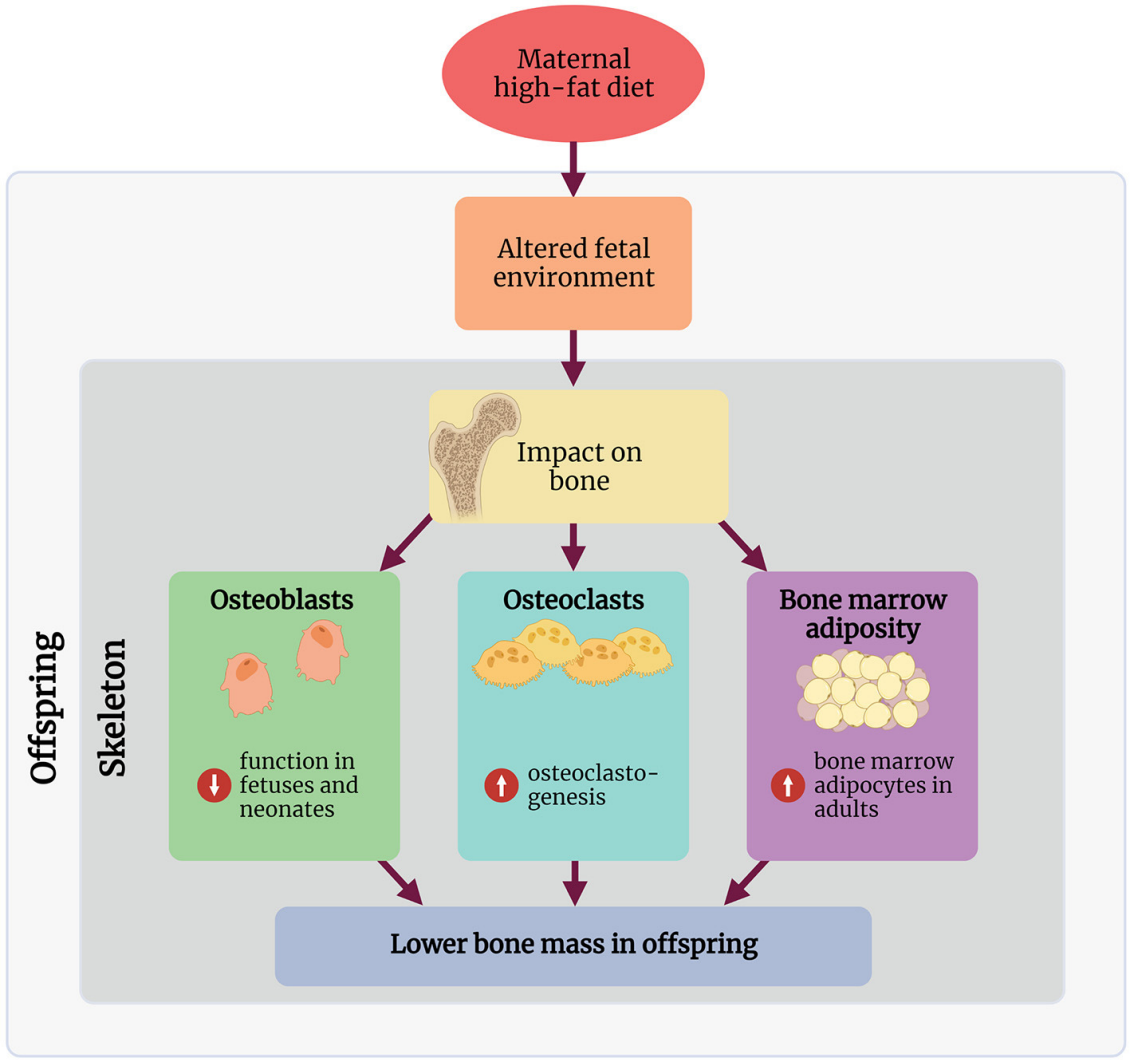
Summary of the proposed mechanisms through which maternal high-fat diet affects the skeleton in the offspring. Created with BioRender.com.

Research into the mechanisms involved in linking maternal HFD with a bone phenotype in rodents remains in its infancy, and to our knowledge, no studies in humans have explored any mechanisms of action. The following section discusses some key mechanisms demonstrated in rodent maternal HFD studies, linking the early life environment and the observed bone phenotype in these offspring (Figure 1).

### Osteoblasts

Osteoblasts are derived from mesenchymal stem cells and are responsible for the synthesis and mineralisation of bone. Whilst osteoblast number is unaffected (22, 24), there may be a negative relationship between osteoblast function in offspring and maternal HFD during pregnancy and lactation. However, this relationship with osteoblast function may be transient and lost as offspring age. Whole-embryo skeletal ossification and total bone volume are decreased following maternal HFD (18, 19). Rat calvarial osteoblasts from offspring exposed to maternal HFD have decreased proliferation and osteoblastic differentiation (19). Therefore, decreased differentiation of osteoblasts could directly be responsible for decreased or delayed bone formation during development.

Although bone marrow stromal cells (BMSCs) are a significant source of osteoblast progenitor cells contributing to bone remodeling, only one study examined whether the differentiation capacity of these cells into osteoblasts is influenced by maternal HFD. Kushwaha et al. assessed cellular activity in 15-week old animals and found no difference in osteogenic differentiation of BMSCs exposed to maternal HFD. However, compared to BMSCs derived from CD-fed mothers, these cells have higher mRNA expression of RANKL, which will have implications for osteoclastogenesis (22).

Osteoblast function in adult rodents exposed to maternal HFD is variable. Circulating osteocalcin concentrations are decreased at 17-weeks of age in mice, indicating osteoblast function is decreased following maternal HFD. Mineral apposition rate (MAR) is a reliable direct measurement of osteoblast function (40); one rat study found no difference in MAR in 15-week old males following maternal HFD (22). Interestingly, Devlin et al. found that MAR is increased in 14-week old male mice exposed to maternal HFD (24). This relationship is no longer detected in male mice at 26-weeks of age (24), indicating the rate of mineralization has decreased to a level similar to maternal CD offspring. Surprisingly, Devlin et al. also found no difference in concentrations of the bone formation marker type 1 procollagen N-terminal (P1NP) in circulation at either 14- or 26-weeks of age, despite observed differences in MAR (24). Overall, there is no consensus on whether maternal HFD affects osteoblast function in adult offspring.

### Osteoclasts

Osteoclasts are multinucleated phagocytic cells responsible for bone resorption and are derived from the macrophage-monocyte cell lineage. Very few studies have considered the effects of maternal HFD on osteoclasts. One study broadly examined *ex vivo* osteoclastogenesis following maternal HFD during pregnancy and lactation (22). Kushwaha et al. demonstrated via histomorphometry that osteoclast number, erosion surface, and osteoclast surface were increased in 15-week old male rats exposed to maternal HFD. *Ex vivo* cultures of osteoclast precursors isolated from these animals had increased potential to differentiate into osteoclasts, with these osteoclasts more numerous and larger. Interestingly, these osteoclasts were more sensitized to the effects of RANKL and had increased RANK mRNA expression. Cultured osteoblasts from these same animals had increased RANKL mRNA expression, indicating that the potential for osteoclastogenesis is increased following maternal HFD. Alternatively, when mice were fed HFD for 6-weeks before mating, pregnancy, and lactation, their offspring demonstrated no significant difference in osteoclast number but decreased osteoclast activity at 14- and 26-weeks of age (24). Increased bone resorption is a major contributor to decreased bone volume that develops when rodents are fed HFD, which is likely secondary to increased inflammation (41). Maternal HFD is known to cause low-grade chronic inflammation in offspring; therefore, it is feasible that this could contribute to bone loss in these animals (42). Chen et al. noted increased inflammatory cytokine production in fetal calvarial osteoblasts exposed to maternal HFD, but this potential mechanism has not been addressed in adult offspring (18). Given these conflicting data, further studies are needed to determine the effects of maternal HFD on osteoclast number and function.

### Bone Marrow Adiposity

The balance between BMSCs giving rise to osteogenic or adipogenic precursors is critical for maintaining bone mass; if this balance is shifted toward adipogenesis, this may come at the expense of osteoblastogenesis (43). Additionally, increased bone-marrow adiposity can affect osteogenesis, with an apparent negative relationship between bone marrow adiposity and bone mass (44). Maternal HFD during pregnancy and lactation (29) or lactation only (28) is associated with increased adipocyte number and adipocyte size in the bone marrow cavity. However, interpretation of these studies is complicated by their study design; offspring were either weaned directly onto HFD or CD, or onto CD followed by HFD between 12 and 24-weeks of age. Both studies had conflicting results as to whether maternal HFD with post-weaning HFD affected bone microarchitecture. Hafner et al. found bone marrow adiposity was increased, and trabecular bone volume was decreased in the maternal HFD/post-weaning HFD group at 24-weeks of age (28). However, Lanham et al. found no difference in bone microarchitecture at 30-weeks of age (29). No studies performed *ex vivo* adipogenesis assays on BMSCs. Further studies are required to confirm whether changes in bone marrow adiposity contribute to the bone phenotype in these offspring.

### Epigenetic Modifications

Epigenetic modifications, including DNA methylation and various post-translational histone modifications, describe changes to gene expression that occur without affecting the underlying DNA sequence. Epigenetic modifications allow the individual to alter gene expression in response to the environment and have long been considered a principal mechanism through which the early-life environment affects offspring (7, 45). Despite this connection, there is a paucity of studies that have specifically measured epigenetic modifications in response to maternal HFD.

Chen et al. demonstrated that maternal HFD promotes cellular senescence in fetal calvarial osteoblasts, potentially suppressing bone formation in the prenatal period in both mice and rats (18–20). These findings were shown to be through increased expression of p300/CBP, which increased H3K27 acetylation, which promoted p53/p21-mediated cell senescence signaling in pre-osteoblasts; increased expression of p300/CPB persisted until adulthood (18). Maternal HFD also promoted increases in methylated CpG sites in the homeobox protein A10 (HoxA10) promoter. HoxA10 is important for fetal osteoblastogenesis and adult bone regeneration.

## Current Research Gaps

Many unanswered questions surrounding how HFD-induced maternal obesity affects bone development and microarchitecture in offspring remain.

One outstanding question is whether the detrimental effects on offspring skeleton are driven by maternal obesity, maternal HFD, or both. In humans, most studies explore the effects of obesity during pregnancy, commonly assessed by measuring body mass index rather than dietary patterns (46). In rodents, the majority of studies implemented a maternal HFD regime at least 4 weeks before mating. This would have induced an obesity phenotype in these dams, as well as ongoing exposure to HFD. However, it is unlikely that maternal obesity would have been induced in studies where HFD-feeding was restricted to pregnancy and lactation, or lactation alone. Therefore, these offspring likely experienced exposure to HFD in the absence of maternal obesity. It is challenging to tease out whether HFD-induced obesity before pregnancy or ongoing maternal HFD affects the skeleton in offspring using a rodent model. Unlike in humans, changing the diet of a rodent from HFD to CD induces rapid weight loss (47). This could be overcome using embryo transfer following pre-conception maternal HFD, placing embryos into CD-fed recipients (46).

Another gap in our understanding is deciphering the effects of maternal HFD on other tissues in offspring and how these effects, in turn, modulate the skeleton. For instance, there is cross-talk between the skeleton and skeletal muscle, adipose tissue, and the endocrine pancreas (48–52). The structure and function of these tissues are affected by the early life environment (7). Therefore, it would be interesting to determine whether this cross-talk is affected by maternal HFD and the downstream effects on the skeleton.

In this mini-review, we exclusively discussed the effect of maternal HFD on the skeleton in offspring; however, other paradigms of early life exposure to nutritional excess are also worthy of exploration. For instance, a maternal high-protein diet (53) and a combination of high-fat and high-sugar diet (54) also negatively impact the skeleton in offspring. Additionally, pre-conception paternal nutrition also has long-term effects on the metabolic health of offspring (55). We are unaware of any studies that have addressed the paternal influence of skeletal development in offspring. Thus, understanding the impact of paternal health will also be necessary for understanding the mechanisms that link the early life environment with skeletal health in offspring.

Critically, we are unaware of any studies investigating the effect of nutritional, pharmacological, or behavioral interventions on skeletal outcomes in the offspring. Whilst we do not yet fully understand the mechanisms that impact the developing skeleton in response to maternal HFD, a significant gap lies in the lack of intervention studies.

## Conclusion

There is growing evidence that exposure to maternal HFD during pregnancy has long-lasting adverse effects on the skeleton of offspring. However, many details surrounding these changes and the mechanisms of action that drive these effects remain unclear, and further basic studies are required. Given the consequences of low bone mass and deranged bone microarchitecture for offspring, advances in our understanding of the developmental origins of bone health is critical in our battle against diseases like osteoporosis.

## Author Contributions

EB designed the review. EB, SB, and MT collected relevant articles. All authors have contributed to writing and revision of the manuscript, read, and approved the submitted version.

## Conflict of Interest

The authors declare that the research was conducted in the absence of any commercial or financial relationships that could be construed as a potential conflict of interest.

## Publisher's Note

All claims expressed in this article are solely those of the authors and do not necessarily represent those of their affiliated organizations, or those of the publisher, the editors and the reviewers. Any product that may be evaluated in this article, or claim that may be made by its manufacturer, is not guaranteed or endorsed by the publisher.

## References

[1] Chooi YC Ding C Magkos F. The epidemiology of obesity. Metab Clin Exp. (2019) 92:6–10. 10.1016/j.metabol.2018.09.005 30253139

[2] Kelly T Yang W Chen C-S Reynolds K He J. Global burden of obesity in 2005 and projections to 2030. Int J Obesity. (2008) 32:1431–7. 10.1038/ijo.2008.102 18607383

[3] Apovia CM. Obesity: definition, comorbidities, causes, and burden. Am J Manag Care. (2016) 22.7:s176–85. 27356115

[4] Davies GA Maxwell C McLeod L Gagnon R Basso M Bos H. Obesity in pregnancy. J Obstetr Gynaecol Canada. (2010) 32:165–73. 10.1016/S1701-2163(16)34432-2 20181319

[5] Sirimi N Goulis DG. Obesity in pregnancy. Hormones. (2010) 9:299–306. 10.14310/horm.2002.1280 21112860

[6] Catalano PM. Obesity, insulin resistance and pregnancy outcome. Reproduction. (2010) 140:365–71. 10.1530/REP-10-0088 20457594 PMC4179873

[7] Warner MJ Ozanne SE. Mechanisms involved in the developmental programming of adulthood disease. Biochem J. (2010) 427:333–47. 10.1042/BJ20091861 20388123

[8] Fernandez-Twinn DS Hjort L Novakovic B Ozanne SE Saffery R. Intrauterine programming of obesity and type 2 diabetes. Diabetologia. (2019) 62:1789–801. 10.1007/s00125-019-4951-9 31451874 PMC6731191

[9] Eriksson JG. Developmental pathways and programming of diabetes: epidemiological aspects. J Endocrinol. (2019) 242:T95–104. 10.1530/JOE-18-0680 30959482

[10] Hur SS Cropley JE Suter CM. Paternal epigenetic programming: evolving metabolic disease risk. J Mol Endocrinol. (2017) 58:R159–68. 10.1530/JME-16-0236 28100703

[11] Vickers MH. Developmental programming and transgenerational transmission of obesity. Ann Nutr Metab. (2014) 64:26–34. 10.1159/000360506 25059803

[12] Drake AJ Liu L. Intergenerational transmission of programmed effects: public health consequences. Trends Endocrinol Metab. (2010) 21:206–13. 10.1016/j.tem.2009.11.006 20005734

[13] Desai M Jellyman JK Han G Beall M Lane RH Ross MG. Maternal obesity and high-fat diet program offspring metabolic syndrome. Am J Obstetr Gynecol. (2014) 211:e1–13. 10.1016/j.ajog.2014.03.025 24631702 PMC4149836

[14] Williams L Seki Y Vuguin PM Charron MJ. Animal models of in utero exposure to a high fat diet: a review. Biochim Biophys Acta. (2014) 1842:507–19. 10.1016/j.bbadis.2013.07.006 23872578 PMC3895417

[15] Boudin E Fijalkowski I Hendrickx G Van Hul W. Genetic control of bone mass. Mol Cell Endocrinol. (2016) 432:3–13. 10.1016/j.mce.2015.12.021 26747728

[16] Warden SJ Roosa SMM Kersh ME Hurd AL Fleisig GS Pandy MG. Physical activity when young provides lifelong benefits to cortical bone size and strength in men. Proc Natl Acad Sci USA. (2014) 111:5337–42. 10.1073/pnas.1321605111 24706816 PMC3986122

[17] Seeman E Martin TJ. Antiresorptive and anabolic agents in the prevention and reversal of bone fragility. Nat Rev Rheumatol. (2019) 15:225–36. 10.1038/s41584-019-0172-3 30755735

[18] Chen J-R Lazarenko OP Zhao H Alund AW Shankar K. Maternal obesity impairs skeletal development in adult offspring. J Endocrinol. (2018) 239:33–47. 10.1530/JOE-18-0244 30307152 PMC6145139

[19] Chen J-R Zhang J Lazarenko OP Kang P Blackburn ML Ronis MJJ. Inhibition of fetal bone development through epigenetic down-regulation of HoxA10 in obese rats fed high-fat diet. FASEB J. (2012) 26:1131–41. 10.1096/fj.11-197822 22131269

[20] Chen J-R Lazarenko OP Blackburn ML Rose S Frye RE Badger TM. Maternal obesity programs senescence signaling and glucose metabolism in osteo-progenitors from rat and human. Endocrinology. (2016) 157:4172–83. 10.1210/en.2016-1408 27653035

[21] Liang C Oest ME Jones JC Prater MR. Gestational high saturated fat diet alters C57BL/6 mouse perinatal skeletal formation. Birth Defects Res. (2009) 86:362–9. 10.1002/bdrb.20204 19750487

[22] Kushwaha P Khambadkone SG Li M Goodman EJ Aravindan N Riddle RC. Maternal high-fat diet induces long-lasting defects in bone structure in rat offspring through enhanced osteoclastogenesis. Calcified Tissue Int. (2021) 108:680–92. 10.1007/s00223-020-00801-4 33386478 PMC8064999

[23] Miotto PM M CL Amoye F LeBlanc PJ Peters SJ Roy BD. Maternal high fat feeding does not have long-lasting effects on body composition and bone health in female and male Wistar rat offspring at young adulthood. Molecules. (2013) 18:15094–109. 10.3390/molecules181215094 24322493 PMC6270313

[24] Devlin MJ Grasemann C Cloutier AM Louis L Alm C Palmert MR. Maternal perinatal diet induces developmental programming of bone architecture. J Endocrinol. (2013) 217:69–81. 10.1530/JOE-12-0403 23503967 PMC3792707

[25] Harasymowicz NS Choi Y-R Wu C-L Iannucci L Tang R Guilak F. Intergenerational transmission of diet-induced obesity, metabolic imbalance, and osteoarthritis in mice. Arthrit Rheumatol. (2020) 72:632–44. 10.1002/art.41147 31646754 PMC7113102

[26] Liang C Oest ME Prater MR. Intrauterine exposure to high saturated fat diet elevates risk of adult-onset chronic diseases in C57BL/6 mice. Birth Defects Res. (2009) 86:377–84. 10.1002/bdrb.20206 19750488

[27] Lanham SA Cagampang FR Oreffo ROC. Maternal high-fat diet and offspring expression levels of vitamin K-dependent proteins. Endocrinology. (2014) 155:4749–61. 10.1210/en.2014-1188 25279792

[28] Hafner H Chang E Carlson Z Zhu A Varghese M Clemente J. Lactational high-fat diet exposure programs metabolic inflammation and bone marrow adiposity in male offspring. Nutrients. (2019) 11:1393. 10.3390/nu11061393 31234301 PMC6628038

[29] Lanham SA Roberts C Hollingworth T Sreekumar R Elahi MM Cagampang FR. Maternal high-fat diet: effects on offspring bone structure. Osteopor Int. (2010) 21:1703–14. 10.1007/s00198-009-1118-4 19936867

[30] Lanham SA Cagampang FR Oreffo ROC. Maternal high fat diet affects offspring's vitamin K-dependent proteins expression levels. PLoS ONE. (2015) 10:e0138730. 10.1371/journal.pone.0138730 26381752 PMC4575216

[31] Kimmel DB Jee WSS. Bone cell kinetics during longitudinal bone growth in the rat. Calcified Tissue Int. (1980) 32:123–33. 10.1007/BF02408531 6773629

[32] Dickinson H Moss TJ Gatford KL Moritz KM Akison L Fullston T. A review of fundamental principles for animal models of DOHaD research: an Australian perspective. J Dev Origins Health Dis. (2016) 7:449–72. 10.1017/S2040174416000477 27689313

[33] Jensen KH Riis KR Abrahamsen B Handel MN. Nutrients, diet, and other factors in prenatal life and bone health in young adults: a systematic review of longitudinal studies. Nutrients. (2020) 12:2866. 10.3390/nu12092866 32961712 PMC7551661

[34] Enstad S Cheema S Thomas R Fichorova RN Martin CR O'Tierney-Ginn P. The impact of maternal obesity and breast milk inflammation on developmental programming of infant growth. Eur J Clin Endocrinol. (2021) 75:180–8. 10.1038/s41430-020-00720-5 32814855 PMC7855210

[35] Harvey NC Javaid MK Arden NK Poole JR Crozier SR Robinson SM. Maternal predictors of neonatal bone size and geometry: the Southampton Women's Survey. J Dev Origins Health Dis. (2010) 1:35–41. 10.1017/S2040174409990055 23750315 PMC3672833

[36] Zhang C Hediger ML Albert PS Grewal J Sciscione A Grobman WA. Association of maternal obesity with longitudinal ultrasonographic measures of fetal growth: findings from the NICHD fetal growth studies–singletons. JAMA Pediatr. (2018) 172:24–31. 10.1001/jamapediatrics.2017.3785 29131898 PMC5808867

[37] Cole ZA Gale CR Javaid MK Robinson SM Law C Boucher BJ. Maternal dietary patterns during pregnancy and childhood bone mass: a longitudinal study. J Bone Mineral Res. (2009) 24:663–8. 10.1359/jbmr.081212 19049331

[38] Yin J Dwyer T Cochrane J Jones G. The association between maternal diet during pregnancy and bone mass of the children at age 16. Eur J Clin Nutr. (2010) 64:131–7. 10.1038/ejcn.2009.117 19756026

[39] Petersen SB Rasmussen MA Olsen SF Vestergaard P Mølgaard C Halldorsson TI. Maternal dietary patterns during pregnancy in relation to offspring forearm fractures: prospective study from the Danish National Birth Cohort. Nutrients. (2015) 7:2382–400. 10.3390/nu7042382 25849947 PMC4425150

[40] Recker RR Kimmel DB Dempster D Weinstein RS Wronski TJ Burr DB. Issues in modern bone histomorphometry. Bone. (2011) 49:955–64. 10.1016/j.bone.2011.07.017 21810491 PMC3274956

[41] Shu L Beier E Sheu T Zhang H Zuscik MJ Puzas EJ. High-fat diet causes bone loss in young mice by promoting osteoclastogenesis through alteration of the bone marrow environment. Calcified Tissue Int. (2015) 96:313–23. 10.1007/s00223-015-9954-z 25673503 PMC4383048

[42] Zhou D Pan YX. Pathophysiological basis for compromised health beyond generations: role of maternal high-fat diet and low-grade chronic inflammation. J Nutr Biochem. (2015) 26:1–8. 10.1016/j.jnutbio.2014.06.011 25440222

[43] Rharass T Lucas S. Mechanisms in endocrinology: bone marrow adiposity and bone, a bad romance? Eur J Endocrinol. (2018) 179:R165–82. 10.1530/EJE-18-0182 30299886

[44] Devlin MJ Rosen CJ. The bone-fat interface: basic and clinical implications of marrow adiposity. Lancet Diabetes Endocrinol. (2015) 3:141–7. 10.1016/S2213-8587(14)70007-5 24731667 PMC4138282

[45] Bansal A Simmons RA. Epigenetics and developmental origins of metabolic dysfunction: correlation or causation? Am J Physiol Endocrinol Metab. (2018) 315:E15–28. 10.1152/ajpendo.00424.2017 29406781 PMC6334998

[46] Christians JK Lennie KI Wild LK Garcha R. Effects of high-fat diets on fetal growth in rodents: a systematic review. Reprod Biol Endocrinol. (2019) 17:39. 10.1186/s12958-019-0482-y 30992002 PMC6469066

[47] Matikainen-Ankney BA Ali MA Miyazaki NL Fry SA Licholai JA Kravitz AV. Weight loss after obesity is associated with increased food motivation and faster weight regain in mice. Obesity. (2020) 28:851–6. 10.1002/oby.22758 32133782 PMC7180106

[48] Ducy P. The role of osteocalcin in the endocrine cross-talk between bone remodelling and energy metabolism. Diabetologia. (2011) 54:1291–7. 10.1007/s00125-011-2155-z 21503740

[49] Sims NA Walsh NC. Intercellular cross-talk among bone cells: new factors and pathways. Curr Osteopor Rep. (2012) 10:109–17. 10.1007/s11914-012-0096-1 22427140

[50] Argiles JM Lopez-Soriano J Almendro V Busquets S López-Soriano FJ. Cross-talk between skeletal muscle and adipose tissue: a link with obesity? Med Res Rev. (2004) 25:49–65. 10.1002/med.20010 15389734

[51] Brotto M Johnson ML. Endocrine crosstalk between muscle and bone. Curr Osteopor Rep. (2014) 12:135–41. 10.1007/s11914-014-0209-0 24667990 PMC4374433

[52] Li F Li Y Duan Y Hu C-AA Tang Y Yin Y. Myokines and adipokines: involvement in the crosstalk between skeletal muscle and adipose tissue. Cytok Growth Fact Rev. (2017) 33:73–82. 10.1016/j.cytogfr.2016.10.003 27765498

[53] Ellur G Sukhdeo SV Khan MT Sharan K. Maternal high protein-diet programs impairment of offspring's bone mass through miR-24-1-5p mediated targeting of SMAD5 in osteoblasts. Cell Mol Life Sci. (2021) 78:1729–44. 10.1007/s00018-020-03608-6 32734584 PMC11071892

[54] Shi Y Saben JL He G Moley KH Long F. Diet-induced metabolic dysregulation in female mice causes osteopenia in adult offspring. J Endocr Soc. (2020) 4:1–14. 10.1210/jendso/bvaa028 32309754 PMC7153749

[55] Soubry A. POHaD: why we should study future fathers. Environ Epigenet. (2018) 4:dvy007. 10.1093/eep/dvy007 29732171 PMC5920283

